# Regulation of Filaggrin, Loricrin, and Involucrin by IL-4, IL-13, IL-17A, IL-22, AHR, and NRF2: Pathogenic Implications in Atopic Dermatitis

**DOI:** 10.3390/ijms21155382

**Published:** 2020-07-29

**Authors:** Masutaka Furue

**Affiliations:** 1 Department of Dermatology, Graduate School of Medical Sciences, Kyushu University, Maidashi 3-1-1, Higashiku, Fukuoka 812-8582, Japan; furue@dermatol.med.kyushu-u.ac.jp; Tel.: +81-92-642-5581; Fax: +81-92-642-5600; 2 Research and Clinical Center for Yusho and Dioxin, Kyushu University, Maidashi 3-1-1, Higashiku, Fukuoka 812-8582, Japan; 3 Division of Skin Surface Sensing, Graduate School of Medical Sciences, Kyushu University, Maidashi 3-1-1, Higashiku, Fukuoka 812-8582, Japan

**Keywords:** atopic dermatitis, skin barrier, filaggrin, filaggrin-2, loricrin, involucrin, IL-4, IL-13, IL-17A, IL-22

## Abstract

Atopic dermatitis (AD) is an eczematous, pruritic skin disorder with extensive barrier dysfunction and elevated interleukin (IL)-4 and IL-13 signatures. The barrier dysfunction correlates with the downregulation of barrier-related molecules such as filaggrin (FLG), loricrin (LOR), and involucrin (IVL). IL-4 and IL-13 potently inhibit the expression of these molecules by activating signal transducer and activator of transcription (STAT)6 and STAT3. In addition to IL-4 and IL-13, IL-22 and IL-17A are probably involved in the barrier dysfunction by inhibiting the expression of these barrier-related molecules. In contrast, natural or medicinal ligands for aryl hydrocarbon receptor (AHR) are potent upregulators of FLG, LOR, and IVL expression. As IL-4, IL-13, IL-22, and IL-17A are all capable of inducing oxidative stress, antioxidative AHR agonists such as coal tar, glyteer, and tapinarof exert particular therapeutic efficacy for AD. These antioxidative AHR ligands are known to activate an antioxidative transcription factor, nuclear factor E2-related factor 2 (NRF2). This article focuses on the mechanisms by which FLG, LOR, and IVL expression is regulated by IL-4, IL-13, IL-22, and IL-17A. The author also summarizes how AHR and NRF2 dual activators exert their beneficial effects in the treatment of AD.

## 1. Introduction

The human epidermis is composed of stratified layers, differentiating from the basal layer on the basement membrane, towards spinous and granular layers, and finally to the outermost cornified layer [1,2,3,4,5,6]. Keratinocytes are the major constituent of epidermal cells. In this stratification process, the keratinocytes undergo well-orchestrated differentiation to optimize the skin barrier to survive the dry, harsh terrestrial conditions. Basal layer keratinocytes, expressing keratin 5 (K5) and K14, divide and move up to the spinous layer. Spinous layer keratinocytes then switch their keratin profile to K1 and K10. Keratins connect to the desmosomes, which are the cell–cell adhesion structures specific for stratified epithelium [1,2,3,4,5,6]. In the granular layer, keratinocytes commit to synthesize keratohyalin granules, which are primarily composed of profilaggrin and loricrin (LOR) [1,2,3,4,5,6]. This process coincides with the decreases of K1 and K10, increase of calcium and activation of protein kinase C [2]. In normal orthokeratotic conditions, cells are denucleated when the uppermost keratinocytes in the granular layer turn into cornified cells (corneocytes) in the cornified layer. Profilaggrin is processed to filaggrin (FLG) repeats and the cleaved N-terminus of profilaggrin translocates into the nucleus and may trigger the denucleation process [7,8,9]. The cornified cells provide a specialized cell membrane called the cornified envelope. The cornified envelope is composed of various cytoskeletal and barrier-related molecules, including K1, K10, desmosomal proteins (envoplakin and periplakin), LOR, FLG, filaggrin-2 (FLG2), and involucrin (IVL), which are crosslinked by transglutaminase 1 and partly by transglutaminases 3 and 5 [1,2,3,4,5,6]. Granular layer keratinocytes also produce lamellar granules, which are rich in polar lipids, glycosphingolipids, free sterols, and phospholipids. The lamellar granule lipids are released and integrated into the intercellular space lipids containing sterols, fatty acids and ceramides in the cornified layer. Some ceramides with ultralong omega-hydroxyl chains are covalently bound to the outside of the cornified envelope via scaffolds of IVL, envoplakin and periplakin [1,2,3,4,5,6]. This thin lipid layer covering the cornified envelope is called the corneocyte-bound lipid envelope [6,10]. These three structures, cornified envelope, corneocyte-bound lipid envelope, and intercellular space lipids, are key players in maintaining appropriate barrier function [1,2,3,4,5,6].

**Figure 1:**
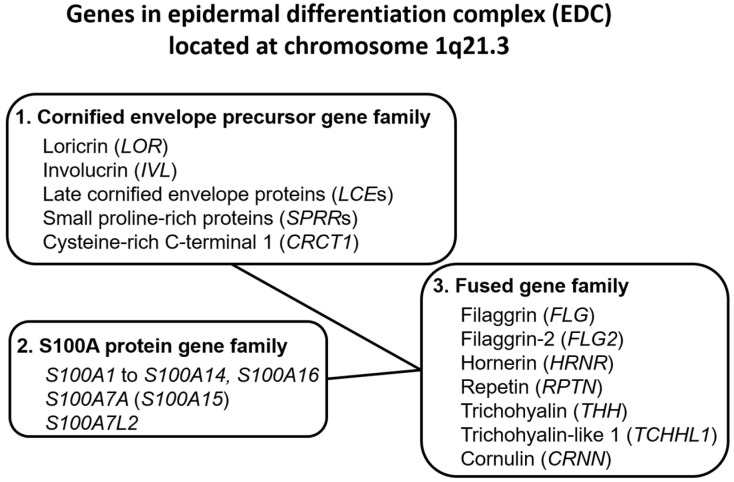
Genes encoding the epidermal differentiation complex.

Upon terminal differentiation, keratinocytes synthesize a number of barrier-related molecules, which are sequentially incorporated into the above-mentioned three cardinal barrier structures. Notably, many barrier-related molecules expressed in the granular layer are genetically mapped to the chromosome 1q21.3 locus, which is called the epidermal differentiation complex (EDC) [5,11]. *FLG*, *FLG2*, *LOR*, and *IVL* genes are all located in the EDC [5,11]. The EDC also includes a series of genes encoding S100A proteins, such as S100A7 [5,12,13]. Most S100A proteins exert antimicrobial and proinflammatory effects [5,12]. *LOR*, *IVL*, late cornified envelope protein genes (*LCE*s), and small proline-rich protein genes (*SPRR*s) are classified into the “cornified envelope precursor gene family” [5]. *FLG*, *FLG2*, and hornerin (*HRNR*) genes are classified into the “fused gene family” evolved from the “S100A protein gene family” and the “cornified envelope precursor gene family” [5] (Figure 1).

The expression of EDC genes is not stable, but is actively modulated by external stimuli, including ultraviolet irradiation and photoproducts [14,15], dioxins, and other oxidative pollutants [4,16], bioproducts of commensal or symbiotic microorganisms such as *Malassezia* and *Staphylococcus epidermidis* [17,18,19], cosmetics [20], and various phytochemicals [21,22,23,24,25]. These chemical stimulants activate the xenobiotic chemical sensor aryl hydrocarbon receptor (AHR), upregulate the expression of barrier-related proteins, and accelerate the terminal differentiation of keratinocytes [4,14,16,18,26]. Consistent with this, long-lasting activation of AHR by dioxins induces the exaggerated terminal differentiation of keratinocytes and sebocytes, leading to the development of chloracne [27,28,29]. In particular, the exaggerated AHR activation converts sebocyte differentiation from sebaceous cell differentiation to keratinocytic differentiation, which results in the loss of sebocytes and keratinous cyst formation [29,30]. This is probably the major cause of chloracne. Although the mechanisms of accelerated keratinization are not fully understood, intracellular levels of reactive oxygen species (ROS) are one of the major modulators [4,31,32,33,34,35,36,37,38]. The intracellular oxidative stress level is fine-tuned by the oxidative stress-prone AHR system [4,31,39] and the antioxidative nuclear factor E2-related factor 2 (NRF2) system [32,33,34,35,36,37,38,40,41,42,43].

The expression of EDC genes is also actively modulated by internal stimuli such as cytokines. The gene expression of *FLG*, *FLG2*, *LOR*, *IVL*, and *S100A7* is differentially affected in atopic dermatitis (AD) and psoriasis [44,45]. The dysregulated expression of *FLG*, *FLG2*, *LOR*, and *IVL* is known to be normalized by specific biologic treatments, for example, blockade of interleukin-4 (IL-4) and IL-13 in AD [44] or blockade of IL-17A in psoriasis [45]. The levels of IL-22 are elevated in both AD [46,47] and psoriasis [48,49]. IL-22 is also known to modulate the gene expression of these barrier-related molecules [50,51,52]. As the expression of *IVL*, *LOR*, *FLG*, *FLG2*, and other EDC genes is differentially altered by these pathogenic cytokines, the expression of these molecules is commonly used as reliable markers for evaluating the therapeutic efficacy of relevant biologics [44,45]. The purpose of this article is to review the current evidence on the regulatory activities of IL-4, IL-13, IL-17A, IL-22, AHR, and NRF2 against IVL, LOR, FLG, and FLG2 gene expression with special reference to AD.

## 2. Roles of IVL, LOR, FLG, and FLG2 in Epidermal Barrier Formation

IVL has high structural homology with LOR in the glutamine- and lysine-rich amino- and carboxy-terminal domains [5]. IVL is expressed in the upper spinous layer, but mainly in the granular layers, and is involved in the initial step of cornified envelope formation. Cornified envelope formation starts from desmosomes where IVL is crosslinked with envoplakin, periplakin, and keratin filaments by transglutaminase 1 [5,6]. This protein complex also becomes the scaffold for the corneocyte-bound lipid envelope [6,10].

LOR is the most abundant component of the cornified envelope [1,3,5]. It is very hydrophobic, insoluble, and is easily polymerized via disulfide crosslinking in ambient air, making it suitable as a protein that reinforces the cornified envelope [1,5]. LOR is expressed in the granular layer and is crosslinked to IVL, envoplakin, and periplakin scaffolds by transglutaminase 1 [1,5].

Profilaggrin consists of a conserved small N-terminal domain, 10–12 FLG repeats and a C-terminal domain [5]. Profilaggrin to FLG processing requires several proteases, such as profilaggrin endopeptidase 1, matriptase 1, and channel-activating protease 1. FLG is involved in aggregating the K1 and K10 filaments into higher-molecular-weight parallel structures that facilitate the incorporation of K1 and K10 into the cornified envelope and contribute to the thin granular keratinocyte shape [1,5,53]. FLG peptides are simultaneously degraded by caspase 14 and calpain 1 into free hydrophilic amino acids, which maintain the intracellular water content [1,5,6]. Ichthyosis vulgaris is caused by the loss-of-function mutation of *FLG* [54]. Loss-of-function mutations of *FLG* have been demonstrated in a subpopulation of patients with AD, at rates ranging from 10% to 50% depending on the ethnicity [55,56]. Therefore, AD is a significant comorbidity with ichthyosis vulgaris [57,58].

FLG2 contains two distinct repeat domains, A and B. The A domain presents high homology with hornerin repeats and the B domain is homologous to FLG [5,59]. FLG2 is also expressed in the keratohyalin granules in the granular layer [5,59]. The expression of *FLG* and *FLG2* is downregulated in skin treated with 5% or 10% lactic acid, with such downregulation often used to define sensitive skin [60]. The expression of FLG and FLG2 has also been reported to be downregulated by tape stripping [61]. In a three-dimensional reconstituted human epidermis model, FLG2 downregulation was found to induce parakeratosis, compact stratum corneum, increased pH, and reduced amounts of free amino acids with reduced proteolytic processing of corneodesmosin, hornerin, and filaggrin in parallel with reduced amounts of caspase-14 [62]. In another report, the expression of FLG2 was described as being colocalized with corneodesmosin in the cornified cells [63]. The absence of FLG2 induces the marked reduction of corneodesmosin expression [63]. Thus, FLG2 exerts a specialized function different from FLG.

Under physiological conditions, IVL is detected from the uppermost spinous layer to granular layer keratinocytes (early-phase epidermal terminal differentiation), but the expression of LOR, FLG, and FLG2 is more confined to granular cell layer keratinocytes (late-phase epidermal terminal differentiation) [5,64,65,66].

## 3. Upregulation of IVL, LOR, and FLG by AHR Activation

**Figure 2:**
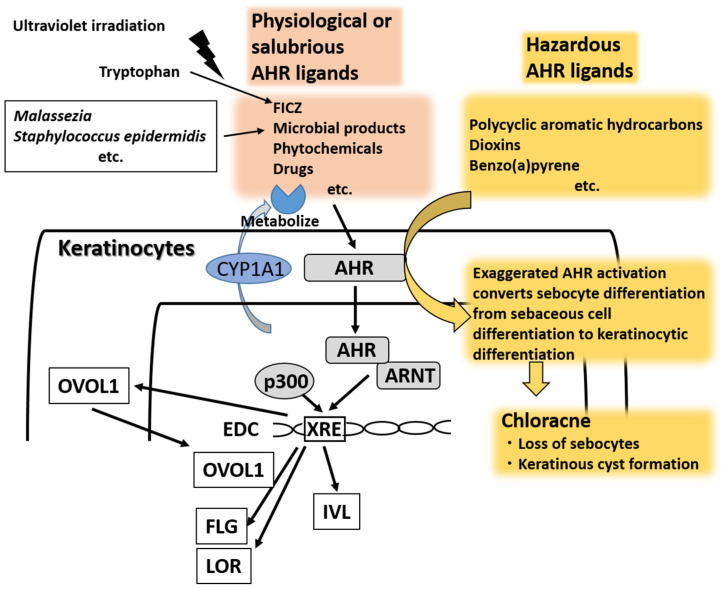
There are many physiological or salubrious aryl hydrocarbon receptor (AHR) ligands such as tryptophan photoproduct 6-formylindolo [3,2-*b*] carbazole (FICZ), microbial products from *Malassezia* and *Staphylococcus epidermidis*, phytochemicals and drugs. AHR activated by ligands translocates into the nucleus and is heterodimerized with AHR-nuclear translocator (ARNT). The ligand–AHR–ARNT complex binds XRE regions with p300 cofactor and upregulates the transcription of target genes and associated protein expression, including for CYP1A1, OVOL1, filaggrin (FLG), loricrin (LOR), and involucrin (IVL). Cytoplasmic OVOL1 translocates into the nucleus and contributes to the upregulation of FLG and LOR, but not that of IVL. The effects of physiological or salubrious AHR ligands are transient because they are rapidly metabolized or degraded by CYP1A1. In contrast, hazardous AHR ligands such as polycyclic aromatic hydrocarbons, dioxins and benzo(a)pyrene are stable and long-lasting in the body because they are not easily metabolized by CYP1A1. The exaggerated AHR activation converts sebocyte differentiation from sebaceous cell differentiation to keratinocytic differentiation. This results in chloracne characterized by the loss of sebocytes and keratinous cyst formation. FICZ, 6-formylindolo [3,2-b] carbazole; XRE, xenobiotic responsive element.

As a chemical sensor, AHR is one of the major transcription factors for EDC genes in keratinocytes. It was originally called dioxin receptor because environmental pollutants such as polycyclic aromatic hydrocarbons and dioxins bind to it with high affinity and generate ROS production [27,28,29,67,68,69]. In the absence of ligands, AHR resides in the cytoplasm, where it forms a protein complex with heat shock protein 90 (HSP90), hepatitis B virus X-associated protein 2 (XAP-2, also known as AIP or Ara9), p23, and c-Src protein kinase [70,71,72]. After ligand binding, AHR dissociates from the cytoplasmic complex and a nuclear translocation site of AHR is exposed. Then, AHR is translocated into the nucleus where it dimerizes with AHR-nuclear translocator (ARNT), binds DNA responsive elements called xenobiotic responsive elements (XREs), and upregulates the transcription of target genes, such as phase I metabolizing enzyme cytochrome P450 (CYP) members (i.e., *CYP1A1*, *CYP1A2*, and *CYP1B1*) [73,74] (Figure 2).

Loertscher et al. were the first to demonstrate that exposure to 2,3,7,8-tetrachlorodibenzo-p-dioxin (TCDD) induces the premature or accelerated terminal differentiation of epidermal keratinocytes with upregulated expression of IVL, LOR, and FLG in three-dimensional skin equivalent models and in vivo models [65,75]. Their findings were later confirmed by Sutter’s group [76]. In monolayer culture, the effects of AHR activation are apparent in human keratinocytes under high-calcium or high-cell-density conditions, in which keratinocytes undergo more differentiation than proliferation [77,78]. In addition, AHR signaling is preferentially activated in differentiated rather than in proliferating keratinocytes [79]. Reciprocally, growth-promoting conditions involving treatment with epidermal growth factor or transforming growth factor α attenuate the TCDD-AHR/ARNT-mediated CYP1A1 and FLG expression [16,78]. The recruitment of nuclear factor p300 plays an essential role in the AHR/ARNT-mediated transcription of target genes [78]. Epidermal growth factor receptor signaling also requires p300 for its proper activity. The competitive usage of p300 by epidermal growth factor receptor activation leads to repression of the recruitment of p300 to the AHR/ARNT transcriptional complex, and eventually suppresses the AHR/ARNT activity to induce transcription of the target gene *CYP1A1* [78] (Figure 2).

Activation of the AHR/ARNT system by TCDD is known to enhance the expression of IVL, LOR, FLG, and FLG2 genes, as well as many other EDC genes, such as hornerin, repetin, small proline-rich protein 2A, late cornified envelope protein 3A, and S100A7 [4,16,65]. The coordinated upregulation of EDC gene products by AHR/ARNT activation highlights the essential involvement of the AHR/ARNT system in epidermal terminal differentiation and skin barrier function. Moreover, AHR antagonists, GNF351 and CH223191, have been shown to inhibit FLG and IVL expression [79]. In addition, TCDD-induced AHR/ARNT activation increases the expression of 75% of the genes required for de novo ceramide biosynthesis, leading to the overproduction of ceramides 1–7 and 9 without affecting the levels of cholesterol and free fatty acids [4]. Moreover, the increased production of the cornified envelope by TCDD is blocked in the presence of antioxidative agents, indicating the important role of oxidative stress in the TCDD-AHR/ARNT-induced acceleration of epidermal terminal differentiation [4]. TCDD-induced ROS production is AHR- and CYP1A1-dependent because TCDD-induced ROS production was shown to be inhibited in AHR-silenced or CYP1A1-silenced cells [80].

Human skin is rich in tryptophan [81,82]. Ultraviolet irradiation generates tryptophan photoproducts such as 6-formylindolo [3,2-*b*] carbazole (FICZ), which is a high-affinity ligand for AHR [83]. This explains why ultraviolet irradiation upregulates the expression of *CYP1A1*, a target gene downstream of AHR/ARNT signaling [84,85,86]. FICZ upregulates the FLG expression in an AHR-dependent manner [15,26].

AHR is a promiscuous receptor and is activated by many other ligands such as bioproducts of commensal or symbiotic microorganisms (*Malassezia* and *Staphylococcus epidermidis*) [17,19,87], cosmetics [20], various phytochemicals [21,22,23,24,25], and drugs [88,89]. These AHR ligands are all known to upregulate the expression of FLG [17,20,22,23,24,25], IVL [17,22], LOR [22,23,24], and transglutaminase 1 [17]. Notably, AHR upregulates the FLG and LOR expression via another transcription factor, OVO-like 1 (OVOL1) [21,22,26,90,91], but OVOL1 is relatively dispensable for AHR-induced IVL upregulation [22] (Figure 2). OVOL1 promotes the differentiation of epidermal keratinocytes by repressing c-Myc-mediated cell proliferation [92,93,94]. Interestingly, the genes encoding FLG, OVOL1, and IL-13 have been reported as the top three genes conferring susceptibility to AD [95].

Under physiological conditions, endogenous AHR ligands, such as tryptophan photoproducts [15,26,83] and microbial bioproducts [17,19,87], may upregulate the expression of EDC genes via AHR activation and maintain the healthy epidermal barrier. In line with this, the expression of FLG, LOR, and IVL is downregulated in keratinocytes with AHR knockdown or in the presence of AHR antagonists during the terminal differentiation of keratinocytes [79]. However, the effects of endogenous AHR ligands are transient because these ligands are rapidly degraded by AHR-induced CYP1A1 [83,96]. In contrast, TCDD and other dioxins are likely to induce long-lasting and exaggerated activation of AHR because these agents are chemically stable and resistant to degradation via CYP1A1 [27,28]. The high-level activation of AHR accelerates the keratinization process of sebocytes and keratinoctytes, leading to chloracne [27,28,29,30,97].

## 4. Decreased Expression of IVL, LOR, and FLG in AD

AD is a common and heterogeneous eczematous skin disorder characterized by Th2-deviated skin inflammation, barrier disruption, and chronic pruritus [98,99,100]. Frequent relapse with intense pruritus deteriorates the quality of life and decreases the treatment satisfaction of afflicted patients [101,102,103,104,105]. Skin barrier dysfunction is associated with the reduced production of terminal differentiation molecules such as FLG [10,31,106,107,108,109].

The expression of INV and LOR was also found to be decreased in the lesional and nonlesional skin of AD [31,110]. Abnormal skin barrier integrity also causes the increased colonization of microbes such as *Staphylococcus aureus,* which further exacerbates Th2-deviated skin inflammation [111,112].

Although the strongest genetic risk factors for AD are loss-of-function mutations in the *FLG* gene [95], *FLG* mutations were not found in all AD patients, were less common in Southern Europeans with AD [113] and were even absent in patients with AD from some African countries [114], suggesting that *FLG* mutations only partly explain FLG protein downregulation in AD. Moreover, FLG mutation was shown not to be related to the development of AD in patients from a subtropical island in Japan [115].

Notably, IL-4 and IL-13 are known to decrease the FLG [20,25,26,31,110,116], LOR [110], and IVL [31,110] expression in vitro. Therefore, a Th2-polarized inflammatory milieu in AD may be more influential in the downregulation of FLG expression than loss-of-function mutation of the *FLG* gene [91,106,117]. The pathogenic importance of IL-4 and IL-13 has recently been reinforced by the excellent treatment response of patients with AD to the anti-IL-4 receptor α (IL-4Rα, *IL4R*) antibody dupilumab, which inhibits both IL-4 and IL-13 signals [44,118]. More recently, large-scale transcriptomic analysis revealed the specific and dominant role of IL-13 in the lesional skin of AD because IL-4 expression was nearly undetectable [119]. Consistent with this notion, the anti-IL-13 antibody tralokinumab was shown to successfully improve AD [120].

In addition to FLG, the expression of LOR, IVL, and FLG2 is downregulated or occurs prematurely in the lesional and nonlesional skin of AD compared with their expression in the normal skin of healthy individuals [26,31,110,121,122,123,124]. In line with these reports, topical steroids significantly improve clinical inflammatory signs and normalize transepidermal water loss in lesional AD skin with the upregulation of FLG and LOR expression [125]. These improvements are associated with downregulation of the Th2 (IL-13 and IL-31) signature [125]. Similar results have been obtained in dupilumab treatment. The expression of FLG and LOR is decreased in the lesional skin compared with that in the nonlesional skin in AD [44]. Dupilumab, but not placebo, restores the downregulation of FLG and LOR [44]. It is also known that the expression of FLG, FLG2, and LOR is downregulated in the patch test site of paraphenylenediamine in patients with allergic contact dermatitis [126].

## 5. Downregulation of IVL, LOR, and FLG by IL-4/IL-13

**Figure 3:**
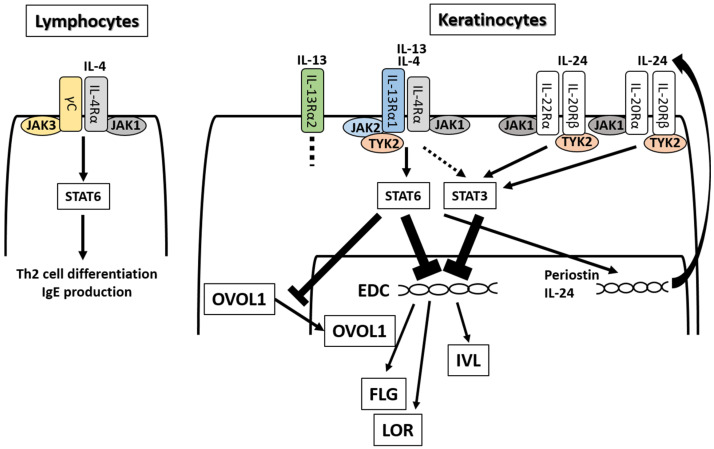
IL-4 and IL-13 have partly shared receptor systems. IL-4 binds IL-4Rα/γC in lymphocytes and other hematopoietic cells, activates the JAK1/JAK3-STAT6 pathway, and induces Th2 differentiation and IgE production. IL-4 and IL-13 share IL-4Rα/IL-13Rα1 in keratinocytes, activate the JAK1/JAK2/TYK2-STAT6 and -STAT3 pathway, and inhibit the expression of EDC molecules such as FLG, LOR, and IVL. IL-4 and IL-13 also inhibit the cytoplasmic-to-nuclear translocation of OVOL1 and downregulate the expression of FLG and LOR. IL-4/IL-13-mediated STAT6 activation upregulates periostin and subsequently enhances IL-24 production. IL-24 binds to IL-20Rβ/IL-22Rα or IL-20Rβ/IL-20Rα, activates the JAK1/TYK2-STAT3 pathway, and inhibits the expression of FLG.

As described above, IL-4Rα signaling by IL-4 and IL-13 is known to reduce the expression of EDC molecules, including IVL [31,110,127,128], LOR [31,127,128], FLG [20,25,26,31,110,116,122,127,128], FLG2 [122,127,128], and hornerin [31,122] in keratinocytes (Figure 3). IL-4 has two heterodimeric receptors (IL-4Rα/γc and IL-4Rα/IL-13Rα1) [129]. IL-13 also has two receptors (IL-4Rα/IL-13Rα1 and IL-13Rα2) [129]. IL-4Rα/γc signaling preferentially occurs in hematopoietic cells, while IL-4Rα/IL-13Rα1 signaling predominantly occurs in nonhematopoietic ones [129,130]. In lymphocytes, IL-4, but not IL-13, induces Th2 cell differentiation and IgE production via IL-4Rα/γc heterodimer [131] (Figure 3). The importance of IL-4Rα/IL-13Rα1 in nonhematopoietic cells coincides with the fact that IL-13, but not IL-4, is preferentially expressed in the lesional skin of AD [44,119]. IL-13Rα2 is a decoy receptor for IL-13 and inhibits IL-13 signaling by trapping functional IL-13 [129,132,133].

After ligation by IL-4, IL-4Rα/γc heterodimer activates Janus kinase 1 (JAK1) and JAK3 and induces the activation (phosphorylation) of signal transducer and activator of transcription (STAT)6 [129,134,135]. IL-4 and IL-13 bind IL-4Rα/IL-13Rα1, activate JAK1, JAK2, and tyrosine kinase 2 (TYK2), and induce the phosphorylation of STAT6 [129,130,136,137]. Although evidence for the STAT6 activation by IL-4 and IL-13 has been consistently found, several groups suggest the possibility that IL-4 and IL-13 activate other STAT family members including STAT1 [128], STAT3 [127,128], and STAT5 [138].

It is still controversial whether IL-4/IL-13-mediated STAT6 activation is fully responsible for the downregulation of EDC molecules [127]. In *Stat6* transgenic mice, the cutaneous expression of *Lor* and *Ivl* was shown to be significantly decreased compared with that in control wild-type mice [110]. In contrast, the permeability barrier function was found to be upregulated in *Stat6*-deficient mice with significantly increased *Lor* expression [139]. Moreover, the *Flg* and *Ivl* expression tended to be upregulated in *Stat6*-deficient mice, but this did not reach statistical significance [139].

Amano et al. highlighted a significant role of the IL-4/IL-13-mediated activation of STAT3, but not STAT6, in the downregulation of FLG and LOR using keratinocytes treated with specific small interfering RNA (siRNA) for STAT3 or STAT6 [127]. IL-4/IL-13-mediated downregulation of FLG and LOR was shown to be canceled in keratinocytes treated with STAT3 siRNA, but not in those with STAT6 siRNA [127]. Notably, IL-4/IL-13-mediated upregulation of CCL26 and CXCL6 expression was canceled in keratinocytes treated with STAT6 siRNA, but not in those with STAT3 siRNA [127]. These results indicate that the IL-4/IL-13-mediated activation of STAT3 transmits signals different from those by the IL-4/IL-13-mediated activation of STAT6, and that IL-4/IL-13-mediated STAT3 activation is probably responsible for the downregulation of EDC molecules in keratinocytes [127]. As both IL-4Rα/γc and IL-4Rα/IL-13Rα1 require the JAK family for their activation, different kinds of JAK inhibitors potently restore the IL-4/IL-13-mediated downregulation of EDC molecules (IVL, LOR, FLG, and FLG2) and improve skin barrier function in vitro and in vivo [127,128].

The IL-13-mediated activation of STAT3 may be caused by another cytokine pathway (Figure 3). IL-4- and IL-13-mediated activation of STAT6 could feasibly upregulate IL-24 production [140,141]. IL-13-induced STAT6 activation upregulates the production of periostin, which promotes allergic inflammation and fibrosis in keratinocytes [142,143]. Periostin stimulates keratinocytes to produce IL-24 [144,145]. IL-24 has two heterodimeric receptors, IL-22Rα/IL-20Rβ and IL-20Rα/IL-20Rβ [146]. After ligation with IL-24, both receptors activate JAK1/TYK2 and STAT3, and downregulate FLG expression [144,145] (Figure 3).

## 6. Medicinal Use of AHR/NRF2 Dual Activators for AD

**Figure 4:**
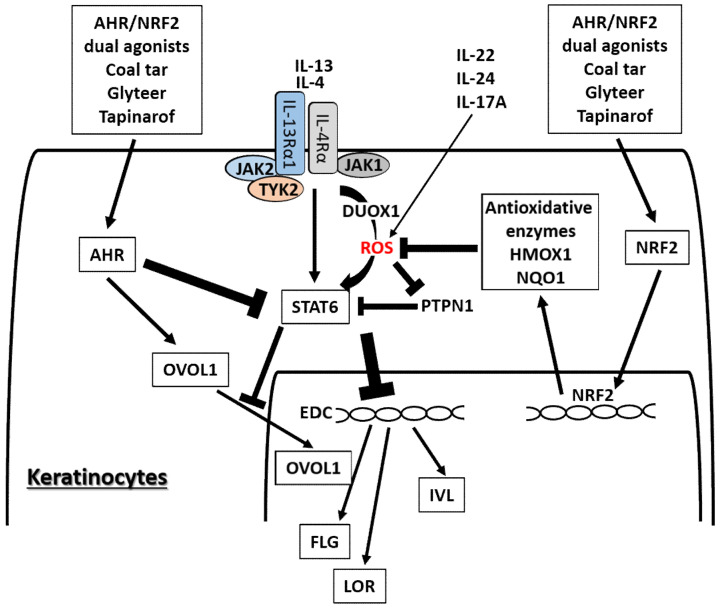
Medicinal AHR/NRF2 dual agonists can restore the IL-4/IL-13-STAT6-induced downregulation of FLG, LOR, and IVL expression. Coal tar and glyteer activate AHR and inhibit the STAT6 effects partially by increasing the entry of OVOL1 into the nucleus. IL-4 and IL-13 activate dual oxidase protein 1 (DUOX1), generate reactive oxygen species (ROS) production, and promote STAT6 phosphorylation in keratinocytes. On the other hand, the activation or phosphorylation of STAT6 by IL-4 and IL-13 is negatively regulated by protein-tyrosine phosphatase, nonreceptor-type 1 (PTPN1) because PTPN1 dephosphorylates the phosphorylated STAT6. ROS induced by IL-4 and IL-13 inhibit PTPN1 activity and subsequently enhance STAT6 phosphorylation. AHR/NRF2 dual agonists also activate NRF2 and upregulate the antioxidative enzymes such as NAD(P)H quinone oxidoreductase 1 (NQO1) and heme oxygenase 1 (HMOX1), which neutralize the IL-4/IL-13-induced ROS and downregulate STAT6 phosphorylation by revitalizing the PTPN1 activity. IL-22, IL-24, and IL-17A also induce ROS production.

Apart from long-lasting and hazardous AHR ligands, many phytochemical AHR ligands play potentially salubrious roles for skin barrier function by upregulating FLG, LOR, and IVL [21,22,23,24,25]. Some AHR ligands are potent NRF2 activators and are expected to be valuable for medicinal use for eczematous skin diseases [21,147] where oxidative stress is upregulated [148,149]. Antioxidative AHR agonists such as coal tar [31], soybean tar glyteer [25,26], and tapinarof [150,151,152] have been widely used or undergone clinical trials for the treatment of inflammatory skin diseases including AD (Figure 4). Coal tar has been integrated into topical skin treatments for more than 2000 years [31]. Glyteer is a delipidated soybean tar licensed in 1924 in Japan and is still covered under the Japanese national medical insurance system as an ointment, in which it is mixed with dexamethasone [25,153]. Tapinarof {5-[(E)-2-phenylethenyl]-2-[propan-2-yl] benzene-1,3-diol, WBI-1001, GSK2894512 or benvitimod} is a naturally derived (but now fully synthetic) hydroxylated stilbene produced by bacterial symbionts of entomopathogenic nematodes [150,152,154,155]. Coal tar, glyteer, and tapinarof could feasibly upregulate the EDC molecules, including FLG, IVL, and hornerin, via AHR activation and exert their antioxidative function via NRF2 activation [25,31,152]. Recent clinical trials of topical tapinarof have proved its efficacy for AD compared with placebo control [150,151,156].

AHR and NRF2 dual activators, coal tar and glyteer, have been shown to restore the IL-4/IL-13-mediated downregulation of IVL, LOR, and FLG [25,31]. IL-4 and IL-13 activate dual oxidase protein 1 (DUOX1), generate ROS production and promote STAT6 phosphorylation in keratinocytes [157] (Figure 4). On the other hand, the activation or phosphorylation of STAT6 by IL-4 and IL-13 is negatively regulated by protein-tyrosine phosphatase, nonreceptor-type 1 (PTPN1) because PTPN1 dephosphorylates the phosphorylated STAT6 [157,158,159]. Oxidative stress induced by IL-4 and IL-13 inhibits PTPN1 activity and subsequently enhances STAT6 phosphorylation [157,158,159]. Coal tar activates NRF2 and upregulates antioxidative enzymes such as NAD(P)H quinone oxidoreductase 1 (*NQO1*), which neutralize the IL-4/IL-13-induced ROS and downregulate STAT6 phosphorylation by revitalizing the PTPN1 activity [31]. The antioxidative AHR agonist glyteer inhibits the IL-4-induced downregulation of FLG, and activates NRF2 and downstream antioxidative enzymes such as NQO1 [24,25]. NRF2 activation also upregulates various other antioxidative enzymes such as heme oxygenase 1 (HMOX1) [40,42,43,143,160] and glutathione peroxidase 2 (GPX2) [161]. As oxidative stress is also capable of enhancing STAT3 activation, it is possible that antioxidative agents may inhibit the STAT3 pathway [162,163].

In parallel with this, other NRF2-activating phytochemicals exhibit similar inhibitory action on IL-4/IL-13-mediated FLG downregulation [20,24], IL-13-mediated periostin upregulation [143] and imiquimod-induced STAT3 activation [164,165] in keratinocytes. IL-4 also stimulates dendritic cells via STAT6 activation to produce CCL17 and CCL22, which are potent chemokines for recruiting Th2 cells [166]. In addition, IL-4 stimulates dendritic cells to upregulate the expression of receptors for IL-31, which is the major pruritogenic cytokine in AD [167]. In addition, antioxidative glyteer inhibits the IL-4/STAT6-mediated expression of CCL17 and CCL22 expression as well as upregulation of the IL-31 receptor [166,167].

OVOL1 is an important transcription factor for epidermal terminal differentiation. It resides in the cytoplasm in a steady-state condition, but activated OVOL1 translocates into the nucleus and regulates downstream gene expression [26,90,92]. OVOL1 suppresses c-Myc expression and inhibits keratinocyte proliferation [93,94], but it conversely promotes epidermal differentiation and upregulates the expression of FLG and LOR [22,26,90]. AHR activation upregulates the expression of OVOL1, induces its cytoplasmic-to-nuclear translocation, and increases the expression of FLG and LOR [22,26,90,92]. IL-4 does not affect or rather enhances OVOL1 expression, but it inhibits the cytoplasmic-to-nuclear translocation of OVOL1, which correlates with the downregulation of FLG expression [26,90]. AHR activation restores the IL-4-mediated inhibition of OVOL1 nuclear translocation and recovers the IL-4-induced FLG downregulation [26,90]. Interestingly, AHR activation also upregulates IVL expression, but its regulation is OVOL1-independent [22]. In addition, IL-4 and IL-13 themselves increase the mRNA and protein expression of AHR in B cells and keratinocytes [168,169]. This suggests a mutually compensatory (or seesaw) regulation between Th2 and AHR signaling. Keratinocytes are a rich source of pro-Th2 cytokines such as IL-33 [98]. AHR-mediated OVOL1 activation is also functional in inhibiting IL-33 production in keratinocytes [170].

## 7. Downregulation of IVL, LOR, and FLG by IL-22

Increased IL-17A and IL-22 signatures are also shown in AD [44,46,47,99,125,171,172,173]. However, the pathogenic roles of Th17 and Th22 cell infiltration have not been fully elucidated in Th2-dominant AD. Guttman-Yassky et al. demonstrated that a blockade of Th2 signaling by dupilumab significantly decreased and normalized not only Th2 signatures, but also Th17 and Th22 signatures, in lesional skin of patients suffering from AD [44]. This suggests the possibility that the increased Th17 and Th22 signatures may be associated with Th2 dominance in AD. Initially, IL-22 was thought to be produced from Th17 cells, but recent human studies have revealed that Th22 cells, which do not produce IL-17A, are the main IL-22 producers [174,175]. Notably, IL-22 production from Th17/22, Th22, and innate lymphoid cells is dependent on AHR [176,177,178,179].

Recent clinical trials of the anti-IL-17A antibody secukinumab, a very potent therapeutic agent for treating psoriasis, did not report its satisfactory efficacy against AD (https://clinicaltrials.gov/ct2/show/results/NCT02594098?term=atopic&cond=secukinumab&draw=2&rank=1). In contrast to IL-17A, IL-22 may exert additional pathogenic effects apart from those mediated by IL-4/IL-13 because the anti-IL-22 antibody fezakinumab shows weak therapeutic potential for treating patients with severe AD [46]. Moreover, fezakinumab is more efficacious for patients with high pretreatment expression of IL-22 than for those with low IL-22 expression [180].

**Figure 5:**
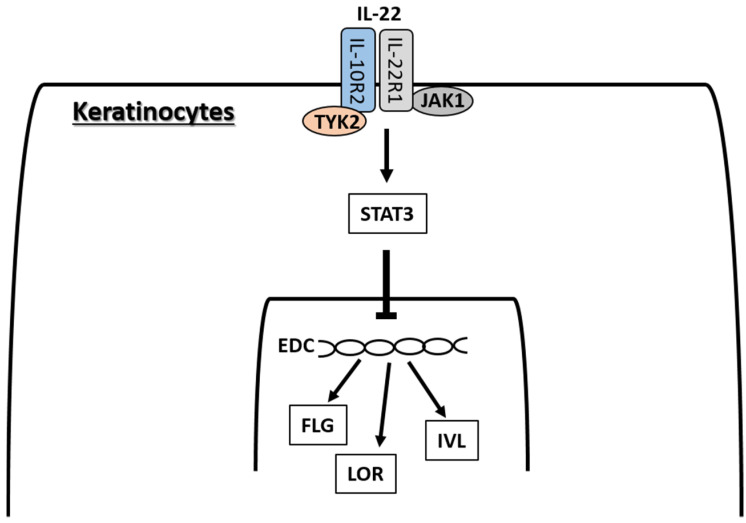
IL-22 binds IL-22R1/IL-10R2 complex, activates the JAK1/TYK2-STAT3 pathway and inhibits the expression of FLG, LOR, and IVL.

IL-22 signaling occurs via a heterodimeric receptor composed of IL-22R1 and IL-10R2, the expression of which is limited to epithelial cells, including those in skin [181,182] (Figure 5). The ligation of IL-22R1/IL-10R2 phosphorylates JAK1 and TYK2, and activates mainly STAT3 in keratinocytes [138,183], and STAT3, to a lesser extent, STAT1 and STAT5 in hepatoma cell lines [184]. IL-22 promotes keratinocyte proliferation and migration in association with STAT3 activation, and inhibits the terminal differentiation of keratinocytes [50,51,138,185,186,187]. IL-22 inhibits the expression of K1 [51,138,185], K10 [50,188], IVL [50,185], LOR [50,188], and FLG [50,51,138,186,188]. House dust mites may enhance the effects of IL-22 because they increase the IL-22R1 expression in keratinocytes [189].

On the other hand, IL-22 upregulates the antimicrobial and proinflammatory EDC molecules, including S100A7, S100A8, and S100A9 in keratinocytes [50]. IL-4 and IL-13 are not significant inducers of S100A7, but its expression is enhanced in the lesional skin of AD probably because IL-17A and IL-22 upregulate S100A7 expression [138,190]. Similar to IL-4 and IL-13 [138,144], IL-22 also upregulates the IL-24 expression, which may inhibit FLG expression via JAK1-STAT3 activation [138,187]. Similar to IL-22, IL-24 is also responsible for keratinocyte proliferation and S100A7 upregulation [52]. Both IL-22 and IL-24 induce ROS production [191,192,193], and antioxidative AHR ligands may ameliorate the inflammatory outcome of IL-22 and IL-24. In fact, the antioxidant luteolin-7-glucoside decreases intracellular ROS levels and inhibits the IL-22-mediated activation of STAT3 [194].

## 8. Regulation of IVL, LOR, and FLG by IL-17A

Although the pathogenic significance of IL-17A is not fully understood in AD, IL-17A plays a critical role in the pathogenesis of psoriasis, as indicated by the excellent efficacy of anti-IL-17A biologics for psoriasis [195,196,197,198,199,200,201,202,203,204,205,206]. IL-17A alone may not be sufficient to fully activate the proinflammatory cascade, but it accelerates psoriatic inflammation with the help of other key pathogenic cytokines such as tumor necrosis factor-α (TNF-α), IL-23, and IL-22 [187,207,208,209].

**Figure 6:**
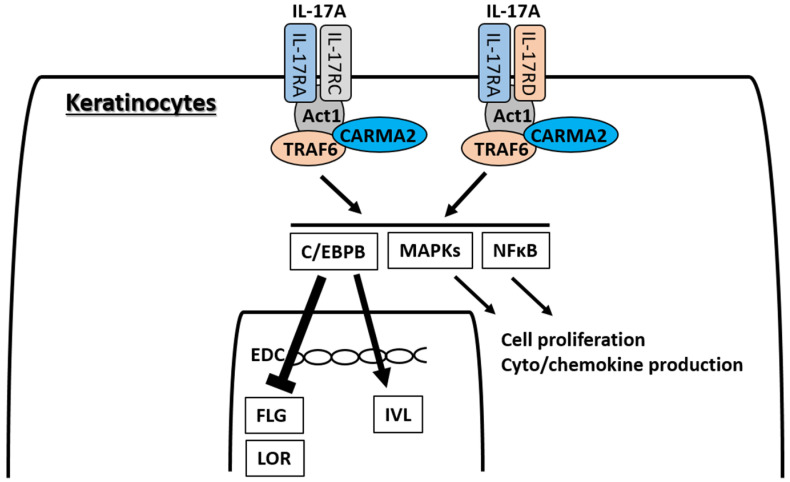
IL-17A has two receptor complexes, IL-17RA/IL-17RC and IL-17RA/IL-17RD, in keratinocytes. Signaling of both receptors occurs via downstream ACT1, TRAF6, and CARMA2 protein complexes, and activates nuclear factor kappa-light-chain-enhancer of activated B cells (NF-κB) and MAPKs. Activation of NF-κB and MAPKs induces cell proliferation and cyto/chemokine production in keratinocytes. IL-17A is not likely to directly activate JAK-STAT pathways. Another important transcription factor for IL-17A signaling is the C/CAAT-enhancer-binding protein β (C/EBPB) or C/EBPD. The IL-17A-C/EBPB pathway is likely to upregulate IVL expression, but to downregulate FLG and LOR expression.

IL-17A signaling is known to occur via two heterodimeric receptors, IL-17RA/IL-17RC and IL-17RA/IL-17RD [210,211,212,213] (Figure 6). Keratinocytes express both IL-17RA/IL-17RC and IL-17RA/IL-17RD, and IL-17A ligation activates the transcription of different sets of genes [213]. Initial subcellular events in the ligation of IL-17RA/IL-17RC by IL-17A are the recruitment and activation of ACT1, TRAF6, and CARMA2 complexes, and the downstream activation of nuclear factor kappa-light-chain-enhancer of activated B cells (NF-κB) and MAPKs [210,211,212,213]. The activation of NF-κB and MAPKs is involved in the IL-17A-mediated keratinocyte proliferation and cyto/chemokine production [208]. The ligation of IL-17RA/IL-17RC by IL-17A induces the activation of NF-κB, ERK, p38 MAPK, and JNK, while that of IL-17RA/IL-17RD mainly activates p38 MAPK and JNK and, to a lesser extent, NF-κB and ERK [213]. IL-17A promotes keratinocyte proliferation directly [208,214] and also indirectly by inducing the production of IL-19 from them [207,215,216,217]. IL-17A is unlikely to directly activate JAK-STAT pathways [138], but may activate STAT3 via IL-19 signaling [218].

Another important transcription factor for IL-17A signaling is the C/CAAT-enhancer-binding proteins (C/EBPs), particularly C/EBPB or C/EBPD [215]. In contrast, IL-17A is likely to inhibit the C/EBPA molecule [214]. The C/EBP family members are involved in epidermal keratinocyte differentiation [219] and are strongly upregulated in the lesional skin of psoriasis [215]. Together with the elevated *CEBPB* gene expression, the expression of keratinocyte terminal differentiation genes, such as *IVL*, *FLG2*, and *TGM1*, is upregulated in the lesional skin of psoriasis [215]. In the promoter region of the *IVL* gene, there is a binding site for C/EBP and the C/EBP transcription factor is necessary for the appropriate and continuous production of IVL protein [220]. In contrast to IVL, IL-17A is reported to downregulate the expression of K10 [51], LOR [51,215], and FLG [138,215,221]. In addition, the expression of S100A7 is upregulated by IL-17A [51,138,215]. Intriguingly, IL-4 and IL-13 are unlikely to affect the S100A7 expression or rather inhibit its expression in keratinocytes [138,222]. Notably, IL-17A is also a potent ROS producer in keratinocytes [192] and recent clinical trials have proven that topical treatment of the antioxidative AHR ligand tapinarof is efficacious for psoriasis [223,224].

## 9. Conclusions

The downregulation of EDC molecules, such as IVL, LOR, FLG, and FLG2, is the cardinal feature of the lesional skin of AD and is associated with skin barrier dysfunction. Although loss-of-function mutation of the *FLG* gene is the genetic abnormality most frequently associated with AD [95], an IL-4- and IL-13-deviated milieu is probably far more important in the pathogenesis of AD when we take into account the excellent efficacy of the anti-IL-4 receptor α antibody dupilumab for AD. IL-4 and IL-13 do inhibit the expression of IVL, LOR, FLG, and FLG2 in keratinocytes in vitro, and the blockade of IL-4 and IL-13 by dupilumab restores the decreased expression of FLG and LOR in the lesional skin of AD.

In addition to IL-4 and IL-13 produced from Th2 cells, IL-22 and IL-17A, produced from Th22 and Th17 cells, are also known to participate in the pathogenesis of AD. IL-4 and IL-13 downregulate the expression of EDC molecules via STAT6 and STAT3 activation. IL-22 also activates STAT3 and inhibits the expression of EDC molecules. IL-22 is probably more potent than IL-17A in downregulating the EDC molecules.

In contrast to cytokine-mediated downregulation, the expression of EDC molecules is upregulated by AHR signaling. In addition to IL-24 [225], IL-4, IL-13, IL-22, and IL-17A are all potent inducers of oxidative stress; therefore, antioxidative AHR ligands with an NRF2-activating profile are expected to be useful for the treatment of AD. Medicinal coal tar and soybean tar glyteer are such AHR and NRF2 dual activators and have been shown to be efficacious in AD. However, these crude agents contain various compounds and have a bad smell. A single chemical compound, tapinarof, is another AHR and NRF2 dual activator, the efficacy for AD of which was recently proven in clinical trials. Of course, exaggerated NRF2 activation seen in the NRF2-transgenic mouse is known to be associated with dysregulated epidermal terminal differentiation [36]. The mechanisms by which EDC molecules are regulated by cytokines, AHR, and NRF2 are not fully understood and there is significant scope for additional investigation. Future studies should open up new strategies for the development of drugs for AD.
